# Outbreak of Severe Zoonotic Vaccinia Virus Infection, Southeastern Brazil

**DOI:** 10.3201/eid2104.140351

**Published:** 2015-04

**Authors:** Jônatas Santos Abrahão, Rafael Kroon Campos, Giliane de Souza Trindade, Flávio Guimarães da Fonseca, Paulo César Peregrino Ferreira, Erna Geessien Kroon

**Affiliations:** Author affiliation: Universidade Federal de Minas Gerais, Belo Horizonte, Brazil

**Keywords:** Vaccinia virus, orthopoxvirus, zoonoses, Brazil, viruses

## Abstract

In 2010, a vaccinia virus isolate caused an atypically severe outbreak that affected humans and cattle in Brazil. Of 26 rural workers affected, 12 were hospitalized. Our data raise questions about the risk factors related to the increasing number and severity of vaccinia virus infections.

After the World Health Organization declared in 1980 that smallpox had been eradicated, smallpox vaccination was suspended (*1*). This fact led to the emergence of a generation of humans that is susceptible to infection by zoonotic viruses of the genus *Orthopoxvirus*, which includes cowpox virus in Europe; monkeypox virus, which occurs naturally in Africa and of which 1 introduction was event reported in the United States; and vaccinia virus (VACV) in Asia and South America (*2*–*5*).

Especially during the past decade, orthopoxvirus (OPV) infections have increased worldwide, and the immunologic status of the population against OPV is a major risk factor for its reemergence (*6*). We describe an outbreak of atypically severe VACV infection in which 12 rural workers in Brazil, who were not vaccinated against smallpox, were hospitalized because of systemic clinical manifestations.

## The Study

In June 2010, an outbreak of exanthematic VACV infection was reported in the rural region of Doresópolis County (20°17′13 ′′S, 45°54′10 ′′ W), Minas Gerais State, Brazil. This region is characterized by small rural properties, where cattle are kept for milk production. Outbreaks of VACV infection had been reported in the neighboring counties in previous dry seasons. In dairy cattle, typical lesions had developed on teats and udders that caused a decrease in milk production; however, the source (index case) was not identified. The reported virulence of the disease in cattle was not atypical and was similar to previously described cases (*4*).

During our collection of epidemiologic data, we were directed to the local health facility, where 12 rural workers were hospitalized because of high fever; lymphadenopathy; prostration; and painful vesicular–pustular lesions on the hands, arms, faces, and/or knees. All patients were occupationally infected (after milking cows that had lesions on teats). Patients reported that in case of multiple lesions, autoinoculation probably occurred from lesions on hands, the first site of infection; therefore, we have no clinical evidence of “generalized vaccinia.” Three patients also had convulsion, vomiting, diarrhea, and mental confusion.

The patients received clinical support and remained hospitalized for 3–18 days. They had no history of immunologic disorders and took no medications that could cause this severe clinical condition. The patients were 15–26 years of age, and none had a history of smallpox vaccination; 1 patient reported having similar clinical illness in 2009. Our investigations also identified 14 additional rural workers who were occupationally infected but not hospitalized; 7 were >40 years old and probably vaccinated against smallpox.

To characterize the etiologic agent of this outbreak, we collected serum from 4 infected cows, scabs from 3 cows, and swab samples from the lesions of 4 hospitalized patients and 1 nonhospitalized patient. The serum samples were submitted to plaque reduction–neutralizing tests as previously described (*7*). Neutralizing antibodies against OPV were detected in 3 (75%) cows; titers ranged from 1:40 to 1:160 neutralizing units/mL. Scabs and swab samples were macerated, and the supernatant, which was diluted 1:100 in phosphate-buffered saline (PBS), was used in a nested PCR for the C11R (viral growth factor) gene, as described previously (*8*,*9*). OPV-specific fragments from all samples were amplified. The samples were also submitted to viral isolation in BSC-40 cells. We isolated the virus from 3 of the nested PCR viral growth factor–positive samples (1each from a cow, a hospitalized patient, and a nonhospitalized patient). All isolates induced the formation of small plaques, similar to group 1 VACV isolates previously identified in Brazil (*4*). After we observed typical poxvirus cytopathic effect, the viruses were plaque-purified and used to reinoculate a Vero cell monolayer for viral amplification.

The viral DNA from the A56R (hemagglutinin) gene was amplified and sequenced from all isolated viruses (*10*). The A56R gene is traditionally used for phylogenetic analysis and usually clusters VACV from Brazil (VACV-BR) into 2 groups (group 1: mice, nonvirulent; group 2: mice, virulent) (*4*). In addition, we sequenced DNA from the A26L viral gene (A-type inclusion body) (*11*). The obtained PCR fragments were directly sequenced in both orientations in triplicate (Mega-BACE 1,000 sequencer; GE Healthcare, Buckinghamshire, UK). The sequences were aligned with previously published OPV sequences from GenBank by using ClustalW (*12*), and the alignments were manually verified by using MEGA 4.0 software (http://www.megasoftware.net). We named these isolates VACV DOR2010 (provisional GenBank accession no. 1606198).

Optimal alignment of the nucleotides from the A56R and A26L genes using ClustalW showed that all amplified DNA sequences from DOR2010 were identical and were highly identical to several group 1 VACV-BR isolates (99.7% identity [A56R] and 99.8% identity [A26L] average) (Figure). DOR2010 also showed a signature deletion of 18 nt in the A56R sequences of other group 1 VACV-BR isolates. Therefore, no special genetic feature was identified in the DOR2010 isolates in regard to A56R and A26L. Phylogenetic trees based on the nucleotide sequences of the A56R and A26L genes of OPV showed that DOR clustered with VACV-BR group 1 (Figure).

**Figure:**
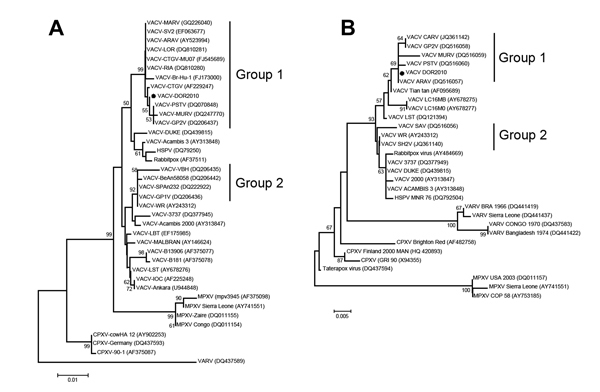
Phylogenetic trees based on the nucleotide sequences of the A56R (A) and A26L (B) genes of orthopoxvirus showing that DOR clusters with Brazilian vaccinia virus(VACV) genogroup 1.The trees were constructed by using the neighbor-joining method and the Tamura-Nei model of nucleotide substitutions with a bootstrap of 1,000 replicates using MEGA 4.0 (http://www.megasoftware.net). Dots highlight VACV DOR2010 among group 1 isolates. GenBank accession numbers appear in parentheses. Scale bars indicate nucleotide substitutions per site.

Given the severity of the outbreak, we investigated the virulence of this isolate in mice (following the rules of the Committee of Ethics for Animal Experimentation, Universidade Federal de Minas Gerais, Belo Horizonte, Brazil). Sixteen BALB/c mice were divided into 4 groups of 4 mice each. We intranasally inoculated 4 mice with 10-μL doses of viral suspensions containing 10^6^ plaque-forming units, as described previously (*4*). Two groups were inoculated with VACV-GuaraniP1 or VACV-GuaraniP2 (*4*), which served as virulent or nonvirulent controls, respectively. Another group was inoculated with PBS. The animals infected with VAVC-GuaraniP1 exhibited ruffled fur, arched backs, and weight loss. No clinical signs were observed in mice inoculated with DOR2010, VACV-GuaraniP2, or PBS, which supported the grouping of the DOR2010 sample into the nonvirulent cluster.

## Conclusions

Our biologic, epidemiologic, and molecular data indicate that the VACV isolate DOR2010 was associated with an outbreak of severe, exanthematous vaccinia virus infection that resulted in the hospitalization of 12 workers in a rural area in Brazil. During the past decade, VACV has spread to all regions of Brazil, and no specific official national programs are in place to prevent the disease (*4*,*13*–*15*). Notifications and scientific efforts are needed to clarify the circulation, virulence, and diversity of VACV. Our data showed that DOR2010 does not present any special feature in A56R or A26L that justifies this unprecedented severity and infectivity among humans. Other studies suggested that group 2 VACV might be associated with severe illness, but such is not the case in the outbreak studied; however, very limited information is available about the relation between viral genotype and virulence in humans. Although useful for pathogenesis studies, our data indicate that the mouse model might not be considered a precise approach to indicate virulence potential of a viral isolate in humans (*4*). Exposure of OPV-nonvaccinated workers to VACV might, in part, explain those clinical features. Although previously infected—and vaccinated—patients were among the patients studied (Table), vaccination history (based on age, vaccine scar, and patient report) was strongly associated with severity of disease. The increased number of VACV outbreaks in recent years should be analyzed in the context of a worldwide phenomenon involving other zoonotic OPVs (*1*–*6*). We believe that the increased number of notifications will be followed by a concomitant increase in reports of atypically severe cases. A worldwide scientific and governmental debate is essential for zoonotic OPV control and prevention on the different continents affected by these viruses.

**Table:** Clinical data of vaccinia virus–infected patients, Brazil, 2010*

Patient no.	Age, y	Signs/symptoms	Reported similar clinical features in previous years	Hospitalization, d	Case definition	Smallpox vaccinated
1	17	F, L, Ls hands, P	No	4	CC, H	No
2	25	F, L, Ls hands/arms, P	No	3	CC, H	No
3	24	F, L, Ls hands/knees, P	No	3	CC, H	No
4	20	F, L, Ls hands, P	No	3	CC, H, LC	No
5	21	F, L, Ls hands/face, P	No	4	CC, H, LC	No
6	21	F, L, Ls hands, P, C, V, D, M	NA	5	C, H	No
7	23	F, L, Ls hands, P	No	7	CC, H, LC	No
8	21	F, L, Ls hands, P, C, V, D, M	No	3	C, H	No
9	18	F, L, Ls hands, P	Yes	10	C, H	No
10	15	F, L, Ls hands/arms, P, C, V, D, M	No	18	CC, H, LC	No
11	26	F, L, Ls hands, P	NA	15	CC, H	No
12	18	F, L, Ls hands, P	NA	6	CC, H	No
13	46	F, L, Ls hands, P	No	No	CC, H	No
14	17	F, L, Ls hands/arms, P	No	No	CC, H	No
15	25	F, L, Ls hands, P	No	No	CC, H	NA
16	28	F, L, Ls hands, P	No	No	CC, H	NA
17	42	F, L, Ls hands/arm, P	No	No	CC, H, LC	NA
18	56	F, L, Ls hands, P	NA	No	CC, H	Yes
19	51	F, L, Ls hands, P	No	No	CC, H	NA
20	62	F, L, Ls hands, P	No	No	CC, H	Yes
21	55	F, L, Ls hands/knee, P	No	No	CC, H	Yes
22	43	F, L, Ls hands, P	NA	No	CC, H	NA
23	19	F, L, Ls hands/arm, P	NA	No	CC, H	No
24	18	F, L, Ls hands, P	NA	No	CC, H	No
25	25	F, L, Ls hands/arms, P	NA	No	CC, H	No
26	26	F, L, Ls hands, P	No	No	CC, H	No

*Data were obtained from the health center and based on observations and patient reports. C, convulsion; CC, clinical confirmation; D, diarrhea; F, fever; H, history of exposure; L, lymphadenopathy; LC, laboratory confirmation; Ls, exhanthematous lesion(s); M, mental confusion; NA, information not available; P, prostration; V, vomiting.
